# Bismuth Pelvic X-Ray Shielding Reduces Radiation Dose Exposure in Pediatric Radiography

**DOI:** 10.1155/2021/9985714

**Published:** 2021-10-11

**Authors:** Bow Wang, Chien-Yi Ting, Cheng-Shih Lai, Yi-Shan Tsai

**Affiliations:** ^1^Department of Diagnostic Radiology, National Cheng Kung University Hospital, College of Medicine, National Cheng Kung University, Tainan 701, Taiwan; ^2^Department of Medical Imaging and Radiology, Shu-Zen Junior College of Medicine and Management, Kaohsiung City 82144, Taiwan; ^3^Association of Radiological Technologists of Tainan City, Tainan 70403, Taiwan

## Abstract

**Background:**

Radiation using conventional X-ray is associated with exposure of radiosensitive organs and typically requires the use of protection. This study is aimed at evaluating the use of bismuth shielding for radiation protection in pediatric pelvic radiography. The effects of the anteroposterior and lateral bismuth shielding were verified by direct measurements at the anatomical position of the gonads.

**Methods:**

Radiation doses were measured using optically stimulated luminescence dosimeters (OSLD) and CIRS ATOM Dosimetry Verification Phantoms. Gonad radiographs were acquired using different shields of varying material (lead, bismuth) and thickness and were compared with radiographs obtained without shielding to examine the effects on image quality and optimal reduction of radiation dose. All images were evaluated separately by three pediatric orthopedic practitioners.

**Results:**

Results showed that conventional lead gonadal shielding reduces radiation doses by 67.45%, whereas dose reduction using one layer of bismuth shielding is 76.38%. The use of two layers of bismuth shielding reduces the dose by 84.01%. Using three and four layers of bismuth shielding reduces dose by 97.33% and 99.34%, respectively. Progressively lower radiation doses can be achieved by increasing the number of bismuth layers. Images obtained using both one and two layers of bismuth shielding provided adequate diagnostic information, but those obtained using three or four layers of bismuth shielding were inadequate for diagnosis.

**Conclusions:**

Bismuth shielding reduces radiation dose exposure providing appropriate protection for children undergoing pelvic radiography. The bismuth shielding material is lighter than lead, making pediatric patients more comfortable and less apt to move, thereby avoiding repeat radiography.

## 1. Introduction

Radiation using conventional X-ray is associated with exposure of radiosensitive organs and typically requires the use of lead for protection. However, lead (Pb) is shown to obscure important anatomical structures during imaging and produce artifacts necessitating repeat radiology [1]. Pelvic radiography, which is one of the more frequent and high-dose examination for children [2], the radiosensitive organs in the lower abdomen, especially the gonads, is exposed to ionizing radiation [3–5]. Because the restlessness or movement of young children during the examination may result in poor organ shielding and reduced diagnostic accuracy, repeat radiography may be needed, which increases the radiation dose, careful consideration of gonadal shielding is warranted. Dose reduction has classically been a significant factor in diagnostic imaging. In pediatric patients, in particular, dose reduction to protect radiosensitive organs is of prim importance. Therefore, effective alternatives to Pb shielding materials are needed to provide adequate radiation protection for radiosensitive organs while maintaining diagnostic accuracy and does not compromise clinical objectives of the examination.

The bismuth shielding material is soft and can be easily placed on the body to shield gonadal areas and other organs such as thyroid gland, colon, lung, stomach in chest, and abdominal area during radiographic examinations. Bismuth has also been evaluated for organ protection in computed tomography (CT) examinations [6–9] and results suggested that it was generally a feasible means of radiation dose reduction depending upon the particular radiographic target. However, few studies have investigated the use of bismuth for radiation dose protection for children who require X-ray-based radiography.

Advantages of bismuth shields have been investigated in two recent studies [3, 4]. Pelvic radiography using bismuth shields in 154 boys and 170 girls demonstrated no detrimental effects on image quality according to European image quality guidelines, and the authors advocated routine use of the bismuth shields [4]. The dosimetry data demonstrated statistically significant dose reductions with use of bismuth shields comparing to nonshielded patients; however, the measured dose exposure was at the surface not at the organ level [3].

We hypothesized dose reduction in gonad tissue is as high as predicted by indirect measurements by Karami et al.; therefore, we designed an experimental study using anthropomorphic cross-sectional dosimetry phantoms equivalent to human tissue and measurement of radiation doses by optically stimulated luminescent dosimetry (OSLD). The study purpose was to evaluate the feasibility of using bismuth shielding for radiation protection in pediatric pelvic radiography; the effects of the anteroposterior and lateral bismuth shielding were verified by direct measurements at the anatomical position of the gonads.

## 2. Materials and Methods

### 2.1. Study Design

This study was designed to investigate the use of bismuth shielding for radiation protection in pediatric pelvic radiography using anthropomorphic cross-sectional dosimetry phantoms equivalent to human tissue. Evaluation of bismuth shielding compared to conventional Pb shielding was intended to demonstrate that X-ray with bismuth shielding produces clear images with a radiation dose benefit that protects target tissue. Dose measurement and image evaluation of lead and bismuth protective shielding materials were compared with results of the control group for which shielding was not used.

### 2.2. Materials

Radiographic examinations in this study were performed using a Philips digital X-ray system (Philips Digital Diagnost System, Philips USA, Andover, MA). A 12-year-old anthropomorphic cross-sectional dosimetry phantoms equivalent to human tissue were used (CIRS ATOM model 701; CIRS, Norfolk, VA, USA). The tissue-equivalent pediatric phantoms are designed to investigate organ doses for specific tissue (e.g., soft tissue, bone, brain, sternum, and lung) or for either whole body effective doses or verification of delivery of therapeutic radiation doses. The OSLDs were used to collect dose data in the radiography range by retaining dosimeter signals after irradiation, as previously described [10, 11]. The OSLDs evaluated were nanoDots dosimeters (Landauer, Inc), and the dosimeters were read using an OSL reader (InLight MicroStar OSL reader (Landauer, Inc., Glenwood, IL)). Before irradiation, the OSLD was placed in the position of the gonad in the ATOM phantom, and the two shielding materials, lead (0.5 mm equivalent lead) and bismuth, were used alternately (one protective bismuth shield is equivalent to 0.06 mm lead protection shield). Fixed exposure conditions of 6.2 mAs and 73 kVp were used to record radiation dose data. Half value layer for Pb was 0.17 mm at 73 kVp, and 0.20 mm for Bi at 73 kVp.

### 2.3. Procedure

**Figure 1:**
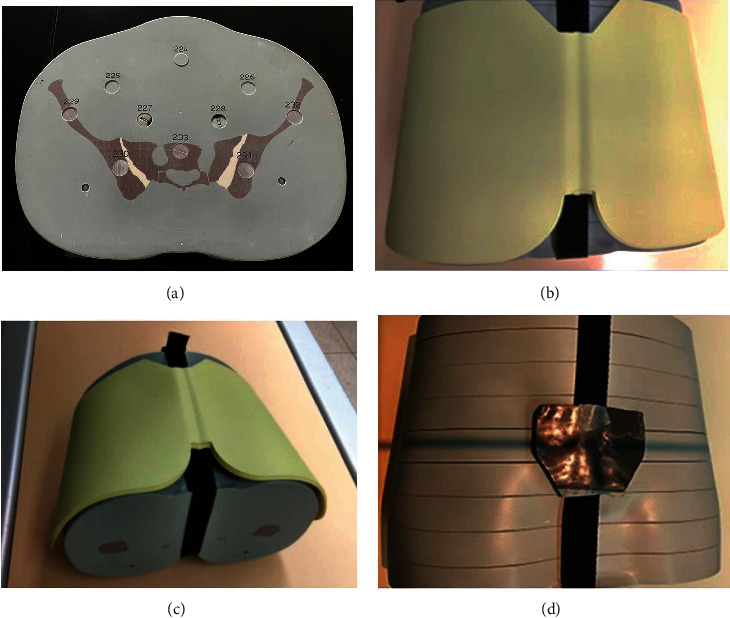
(a) OSLD chip is placed in the position of number 227 and 228 in the prosthesis to measure the radiation dose of the tissue organ. (b) Front bismuth shielding. (c) Front and side bismuth shielding. (d) Lead shielding.

The procedural steps were as follows: (1) place the OSLD at the normal anatomical position of the gonad in the phantom and irradiate the phantom without using any protective shielding. (2) Use the same exposure conditions with conventional Pb shielding (0.5 mm equivalent lead) to evaluate the radiation doses received by the gonad. (3) Use one to four layers of bismuth shielding to cover the front and sides of the body completely to evaluate the radiation dose received by the gonad while using bismuth shielding (Figure 1). (4) Repeat step 3 but cover only the front of the body with bismuth shielding. Covering the front and side of the body was done to mainly understand differences in scattered radiation received on the side of the body. The above experimental procedure was repeated three times, and the measurements and images were collected.

### 2.4. Defining Image Quality

**Figure 2:**
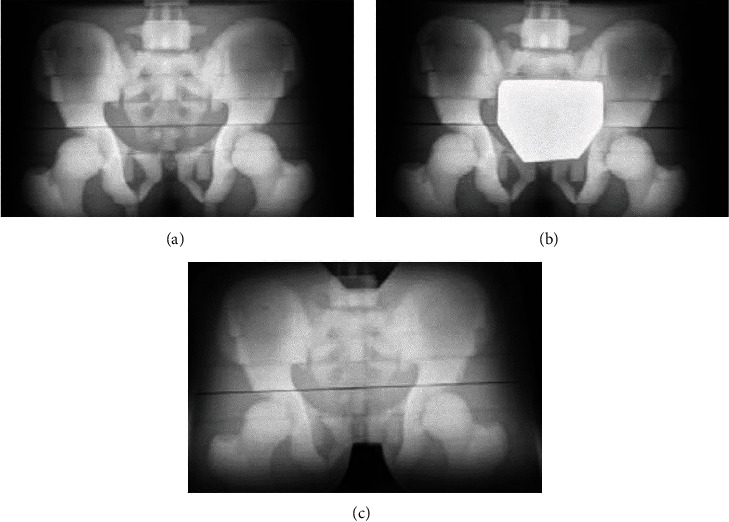
Representative X-ray images from different shielding materials: (a) radiography without any protection, (b) using conventional lead shielding tablets, and (c) using 4-layer bismuth shielding tablets; front and side shielding.

**Figure 3:**
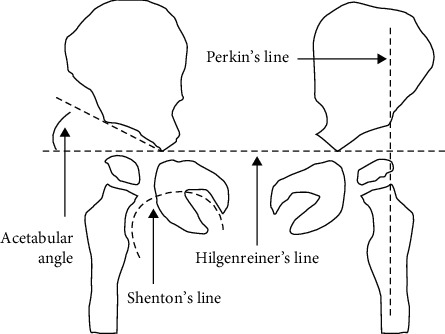
Image quality. The image must be clear enough to measure the following four lines: Perkin's line, Hilgenreiner's line, Shenton's line, acetabular angle.

Images obtained with bismuth shielding were evaluated for quality (Figure 2). Two radiologists and one orthopedic physician evaluated the images and interpreted the result base on the standard used for making clinical diagnosis. The number of clear image line visible in each condition was recorded. Data were analyzed statistically to determine whether the images were in accord with the clinical diagnosis. Image interpretation included (1) Hilgenreiner (H line) clarity, (2) acetabular top angle (acetabular roof angle) sharpness, (3) P line (Perkin) clarity, and (4) S Shenton (S line) clarity (Figure 3). These lines were selected because they measure the area to assess hip dysplasia (e.g., developmental dysplasia of the hip). The images with highest quality were selected according to statistical analysis.

### 2.5. Statistical Analysis

Radiation doses were measured three times and the final values were expressed as mean ± standard deviation. The dose ratio was compared between the bismuth protected group and the Pb-shielded group and nonprotected controls. Dose ratio was calculated as (mean unprotected dose value − mean protective dose value) / mean unprotected dose value.

## 3. Results

The mean radiation dose exposure of the gonadal area without the use of protection was 149.29 uSv, and mean dose exposure using 0.5 mm Pb protection shield was 48.60 uSv, a reduction of 67.45% in exposure dose compared with that without radiation protection. The mean dose exposure with one protective bismuth shield covering the gonadal area was 35.26 uSv (Table 1).

Results showed that bismuth shielding is associated with a lower radiation dose exposure than Pb shielding. The dose measured when one to four bismuth shields was used to cover the anteroposterior of the gonad ranged from 35.26 uSv to 0.99 uSv, representing a reduction of 76.38% to 99.34% compared with the unprotected control, and a dose reduction of 27.45% to 97.96% compared with Pb protection (Table 1).

The dose measured when one to four bismuth shield to cover the anteroposterior and lateral (AP and lateral) of the gonad ranged between 39.03 uSv and 3.47 uSV, representing a reduction of 73.85% to 97.68% compared with the unprotected control and 19.69% to 92.86% compared with Pb protection (Table 1).

### 3.1. Image Quality

The image quality is shown in Table 2. All three physicians agreed that the image quality of the bismuth shielding image conformed to the clinical requirements for diagnosis. However, when three bismuth shielding layers were used, two physicians thought that the images were not clear enough to clearly distinguish all measured lines. Additionally, all three physicians concluded that using four bismuth shielding layers produced poor image quality.

## 4. Discussion

The present study has demonstrated that bismuth shielding is a feasible alternative to Pb shielding for pediatric pelvic radiation protection. Results showed that bismuth shielding is associated with a lower radiation dose exposure even when the layer thickness of bismuth is thinner than the layer thickness of Pb. In addition, the bismuth material is softer and lighter than lead, which is more comfortable for patients and may help to reduce the restlessness and movement that interfere with image quality, as well as to reduce the need for repeat radiography.

The use of phantom human tissue simulation in our study improves the position of measuring dose exposure in a different manner than the conventional pelvic shielded radiography procedure [1]. Use of the phantom human tissue allows the radiation dosimeter to be placed close to the organ of interest, directly simulating the exposure at the position of the gonad/ovary.

In a recent meta-analysis of studies that together included 237 human subjects and 34 pediatric and adult phantoms, the efficacy of bismuth shielding in reducing radiation dose was influenced by several factors, including CT scanner type, use of foams, beam energies, backscatter radiation, and image quality [12]. Nevertheless, a high percentage (89%) of studies in that meta-analysis recommended bismuth shielding based on maintenance of image quality under shielding. The effectiveness of bismuth shielding was also evaluated for its radiation protection ability in pediatric head radiography, specifically for the eyes and thyroid [13–15]. A Slovakian study verified the effectiveness of bismuth shields for radiation protection of the eyes and thyroid in CT exams, which had increased markedly in the last decade in that country; radiation load was decreased in the majority of procedures in several clinical departments, although the authors emphasized that establishment of correct exposure settings was essential in supporting such reductions [13]. Matsutomo et al. [14] found that bismuth-coated latex shields decreased radiation doses of brain SPECT/CT by about 60% without changing attenuation correction or radioactivity concentration. In a study that evaluated a series of phantoms using different thicknesses of shields with both Pb and bismuth demonstrated that a small thickness of bismuth (0.2 mm) or lead shield (0.4 mm) caused significant reduction in absorbed dose and concluded the presence of shield did not affect image quality if superficial organs are not the target of CT imaging and that bismuth or lead shielding technique is an useful and valuable tool in CT to reduce radiation risk in children [16].

In the present study, when two bismuth shielding layers were used to protect the front of the gonadal area, the image quality was sufficient to meet clinical needs and reduce the radiation dose by 81.06%. When shielding both the front and sides of the gonad, 84.01% dose reduction was noted. Therefore, fewer scattered rays were found on the other side of the gonad, meaning that a small difference is found in radiation protection between the sides of the body when multiple shields are used. Theoretically, the use of high Pb equivalent protective equipment has better protective ability; however, in our experience, bismuth provided even more effective protection than did Pb. This could be because the atomic mass of bismuth is greater than that of Pb, and the *K* absorption edge of bismuth (90.5 keV) generated by the binding energy is more extensive than that of Pb (88.0 keV) [17], the number of photons blocked is relatively large. In the present study, when the bismuth shielding tablets were tested for protecting the front of the body and the front and sides of the body, the dose measured at the two ovaries did not differ considerably, because the dose was mainly incident from the direction of the main X-rays; hence, shielding the front of the body blocks most of the dose, and the scattered dose produced on both sides of the body is relatively small. In pelvic radiology, positioning of the automatic exposure-control (AEC) chambers, pelvic orientation, and gonadal shielding can potentially optimize the radiation dose and the image quality [5, 18]. However, those authors concluded that using gonadal shielding can increase the dose area product, leading to repeated radiography exams, especially in female examinations, which raises the question of the actual utility of gonadal shielding in female pelvic radiography. Other authors suggest that the disadvantages of gonadal shielding in pelvic radiography outweigh the benefits, citing mainly that the reduction of risk is negligibly small when considering the possible loss of diagnostic information, repeat radiography, and shielding of AEC chambers [19]. A 2020 study of gonad shielding by Jeukens et al. [20] concluded that when using modern, optimized X-ray systems, gonad shielding can be safely discontinued in females and have marginal benefits in males, justifying discontinuation.

Considering the studies cited above, clearly not all clinicians or all radiologists agree that radiation shielding is necessary. In 2019, the American Association of Physicists in Medicine (AAPM) issued a Statement on the Use of Patient Gonadal and Fetal Shielding (available at http://aapm.org/org/policies/details.asp), which claimed that gonadal and fetal shielding provide negligible, or no, benefit to patients' health and, in fact, “are not associated with measurable harm to the gonads or fetus.” In addition, according to the American College of Radiology (ACR) and the AAPM, shielding affects exposure control and image quality negatively [21]. Shielding with bismuth in systems with automatic-exposure controls was considered to lead to unpredictable and undesirable dose levels and image quality. However, while the AAPM position statement suggests that patient gonadal and fetal shielding during X-ray-based diagnostic imaging should be discontinued as routine practice, the suggestion was mainly for adult patients. Judicious consideration of gonadal shielding is warranted for pediatric patients, because children are 2 to 15 times more radiosensitive to radiation compared to adults [22–24] and have greater risk for experiencing delayed radiation effects [24]. Protection of radiosensitive organs is necessary, and radiation dose reduction is of prim importance in pediatric diagnostic imaging to help protect the sensitive organs during growth and development of the pediatric subjects. Results of the present study suggest that bismuth is very useful in this regard, especially to provide clear images for diagnostic accuracy, and to help avoid repeated radiographic examinations of young children in need of protection to radiations.

This experimental study is a preliminary study of the effectiveness of bismuth as a shielding material for pelvic radiography examinations in infants and children and was conducted using phantom equivalents of human tissue. Limitations may include the lack of human subjects, but in an initial assessment of the protective properties of bismuth, the use of phantom pediatric human equivalents resulted in more objective dose-effect measurements. Further prospective study in human subjects is warranted to confirm results of the present study.

## 5. Conclusions

Our use of the pediatric phantom body allowed us to evaluate different conditions in the thickness of bismuth shielding applications to achieve our goal of reducing radiation exposure while maintaining the optimum image quality for diagnostic accuracy. We found bismuth shielding is associated with lower radiation dose exposure than lead shielding and provides appropriate radiation protection for children undergoing pelvic radiography examination. The bismuth shielding material is lighter than lead, which makes the patient more comfortable and less prone to movement due to discomfort during examination. Further research is needed to ascertain the effectiveness of bismuth radioprotective shielding.

## Figures and Tables

**Table 1:** Dose exposure changes with the use of different protective materials shielded at different tissue locations.

Shielding position	Shielding type	Dose exposure (uSv)	Dose reduction shielding vs. no-shielding	Dose reduction Bi vs. Pb shielding
Unprotected	No protection	149.29 ± 0.95	NA	NA
Ovary	Pb (0.5 mm equivalent Pb)	48.60 ± 0.74	67.45%	NA
Gonad AP	Bi (0.06 mm equivalent Pb)	35.26 ± 0.09	76.38%	27.45%
	Bi (0.12 mm equivalent Pb)	23.88 ± 0	84.01%	50.87%
	Bi (0.18 mm equivalent Pb)	3.99 ± 0.05	97.33%	91.80%
	Bi (0.24 mm equivalent Pb)	0.99 ± 0.04	99.34%	97.96%
Gonad AP and lateral	Bi (0.06 mm equivalent Pb)	39.03 ± 0.81	73.85%	19.69%
	Bi (0.12 mm equivalent Pb)	28.28 ± 0.63	81.06%	41.81%
	Bi (0.18 mm equivalent Pb)	20.16 ± 0.56	86.50%	58.52%
	Bi (0.24 mm equivalent Pb)	3.47 ± 0.05	97.68%	92.86%

Dose exposure is expressed as mean ± standard deviation. Dose Reduction% = (mean unprotected dose – mean dose with protection) / mean unprotected dose. AP: anteroposterior; Bi: bismuth, Pb: lead.

**Table 2:** The number of clear image lines visible when using Bi shields of different thicknesses.

	First doctor	Second doctor	Third doctor
Unprotected	4	4	4
Bi (0.06 mm equivalent Pb)	4	4	4
Bi (0.12 mm equivalent Pb)	4	4	4
Bi (0.18 mm equivalent Pb)	3	3	4
Bi (0.24 mm equivalent Pb)	2	2	2

Bi: bismuth; Pb: lead.

## Data Availability

The data used to support the findings of this study are included within the article.

## Additional Points

*Research Involving Human Participants and/or Animals*. This study was designed to investigate the use of bismuth shielding for radiation protection in pediatric pelvic radiography using anthropomorphic cross-sectional dosimetry phantoms equivalent to human tissue. Therefore, no human participants or animals involved in the study.
